# Effect of intermittent theta burst stimulation on upper limb function in stroke patients: a systematic review and meta-analysis

**DOI:** 10.3389/fneur.2024.1450435

**Published:** 2024-10-11

**Authors:** Junyue Lu, Jiahao Huang, Anqi Ye, Chen Xie, Pan Bu, Jiliang Kang, Jiaxuan Hu, Youliang Wen, Haoyuan Huang

**Affiliations:** ^1^School of Rehabilitation Medicine, Gannan Medical University, Ganzhou, China; ^2^Third Affiliated Hospital of Gannan Medical University, Ganzhou, China

**Keywords:** stroke, upper limb function, iTBS, meta-analysis, systematic review

## Abstract

**Background:**

Stroke is a serious health issue that affects individuals, families, and society. Particularly, the upper limb dysfunction caused by stroke significantly reduces the quality of life for patients and may lead to psychological issues. Current treatment modalities are not fully effective in helping patients regain upper limb motor function to optimal levels. Therefore, there is an urgent need to explore new rehabilitation methods to address this issue.

**Objective:**

The purpose of this meta-analysis and systematic review is to explore the effects of intermittent theta burst stimulation (iTBS) on upper limb function in stroke patients.

**Methods:**

We searched PubMed, Cochrane Library, Embase, Web of Science, PEDro and China National Knowledge Internet as of April 8, 2024. Retrieved a total of 100 articles. Standardized mean differences (SMDs) and 95% confidence intervals (CI) were calculated.

**Results:**

The study included a total of 9 trials and involved 224 patients. The results demonstrate that compared to the control group, iTBS therapy significantly improved Fugl-Meyer assessment-upper extremity (FMA-UE) scores (SMD = 0.88; 95% CI = 0.11–1.66; *P* = 0.03, *I*^2^ = 84%), Action Research Arm Test (ARAT) scores (SMD = 0.83; 95% CI = 0.16–1.50; *P* = 0.02, *I*^2^ = 57%), and Barthel Index (BI) scores (SMD = 0.93; 95% CI = 0.53–1.32; *P* < 0.0001, *I*^2^ = 0%) in stroke patients.

**Conclusions:**

The comprehensive evidence suggests that iTBS has superior effects in improving upper limb function and activities of daily living in stroke patients.

## 1 Introduction

Stroke is a cerebrovascular disease with a high incidence, mortality, and disability rate, being one of the leading causes of disability and death (1). From 1990 to 2019, the incidence of stroke increased by 70.0%, the mortality rate by 43.0%, and the prevalence by 102.0% (2). Currently, stroke has become a serious global public health issue. The most common impairment after a stroke is motor dysfunction, common upper limb impairments include paralysis, loss of fine motor skills, abnormal muscle tone, or changes in somatosensorial (3). Affecting over 80% of stroke survivors with contralateral hemiparesis (4). This significantly impacts their quality of life, functional independence, work, and social interactions (5). Patients may struggle to use utensils like knives and forks effectively, making eating difficult and even requiring assistance from others. Inability to dress independently, including tying shoelaces, further complicates their daily life. These challenges can also have negative psychological impacts, such as decreased self-esteem, anxiety, and depression, as they may hinder participation in social activities and independent completion of daily tasks, ultimately affecting the patients' quality of life and mental wellbeing.

Despite advancements in stroke rehabilitation, achieving meaningful recovery of upper limb function remains challenging for many stroke survivors (6). Although there is substantial evidence supporting the use of conventional therapies such as intensive exercise training or constraint-induced movement therapy for improving upper limb function, they often have limited efficacy for most patients. This limitation may stem from the high costs associated with long-term care and treatment, or the presence of complex physiological and neurological factors unique to each patient, which can influence the effectiveness of treatment. As a result, these approaches are typically insufficient to restore full autonomy. Therefore, there is now a need for novel and effective rehabilitation methods (7).

Neuronal plasticity plays a pivotal role in the rehabilitation of neurological injuries, potentially serving as a key in treating post-stroke motor dysfunction (8). Neurostimulation intervention has shown great potential in the treatment of post-stroke motor impairments, cognitive deficits, and other related disorders in the field of neurorehabilitation (9). Transcranial magnetic stimulation (TMS) is a form of neurostimulation intervention that induces transient electrical currents in target brain regions by applying adjustable-frequency magnetic stimulation. This process depolarizes neurons in the targeted area, aiming to achieve therapeutic effects (10). Among them intermittent theta burst stimulation (iTBS) is a specific pattern of TMS, characterized by its heightened specificity and precision. It involves the application of short bursts of magnetic field stimulation at a high frequency (typically 50 Hz) repeated over the course of ~2 s, followed by a rest period, and then repeated for a total duration of ~190 s (11, 12). The working principle of iTBS is based on the theory of long-term potentiation (LTP) and long-term depression (LTD), which are fundamental mechanisms of synaptic plasticity in the brain. By mimicking naturally occurring patterns of neuronal activity, iTBS can facilitate LTP, enhancing synaptic transmission and connectivity between neurons, thereby enhancing brain function (13). Additionally, iTBS can also inhibit LTD, reducing the weakening of synaptic transmission and helping to prevent the decay of undesirable neuronal connections. Therefore, iTBS is considered an effective neurostimulation intervention that can be used to improve various neurological disorders. Recent studies have demonstrated that intermittent Theta Burst Stimulation (iTBS) is more effective than a placebo in enhancing upper limb motor function, establishing it as the preferred stimulation protocol for both the acute and subacute stages of stroke (14). Furthermore, research has explored whether combining iTBS with other treatments would lead to greater improvement in upper limb function among stroke patients. For instance, studies have investigated combining iTBS with robot-assisted training or integrating iTBS with virtual reality technology (15, 16). However, the impact on upper limb motor function has not been firmly established.

The advantages of iTBS include its significantly shorter stimulation duration compared to traditional high frequency Repetitive transcranial magnetic stimulation (rTMS) (17). iTBS employs intermittent theta-burst stimulation, completing a session in just 2–3 min, whereas high-frequency rTMS generally requires 20–40 min (18). The reduced stimulation time with iTBS allows patients to finish treatment more quickly, thereby decreasing overall treatment time. Additionally, the lower stimulation intensity of iTBS enhances patient acceptability of the treatment. iTBS is indeed recognized as effective for treating a variety of neuropsychiatric disorders, such as depression and anxiety (19). This technique effectively activates specific brain regions, and its stimulation parameters offer high flexibility, allowing adjustments according to individual patient needs (20). This personalized approach makes iTBS a powerful tool, enabling optimization of treatment based on the unique circumstances of each patient. iTBS generally has milder side effects, these side effects, including headaches, localized skin discomfort (such as irritation), and slight dizziness, typically resolve quickly after treatment (21). Compared to high frequency rTMS (such as continuous stimulation at 10 Hz or higher), iTBS has a shorter pulse sequence and its intermittent stimulation pattern generally results in fewer side effects, thereby reducing discomfort (22).

This study aims to provide robust evidence for clinical practice by conducting a systematic review and meta-analysis to assess the impact of intermittent theta burst stimulation (iTBS) on upper limb function in stroke patients.

## 2 Methods

### 2.1 Protocol and registration

This meta-analysis was strictly designed and conducted in accordance with the Cochrane Handbook and the PRISMA guidelines (23, 24). And it was registered with PROSPERO (CRD42024525157). The data included in this study all come from experimental articles, with no direct contact with patients, thus this study complies with the principles of experimental ethics.

### 2.2 Literature search

A comprehensive search was conducted on six databases, including PubMed, The Cochrane Library, Embase, Web of Science, Pedro, and China National Knowledge Infrastructure (CNKI). The search was conducted from the establishment of these databases until April 8, 2024. The databases were searched for experimental literature on the use of iTBS intervention in stroke patients with upper limb dysfunction. There were no language restrictions applied during the search process. The search terms were “Intermittent theta burst stimulation,” “stroke,” and “upper limb function.” Two researchers (Lu and JH Huang) independently conducted the research. If any disagreements arise during this process, a third researcher (HY Huang) will review and make the final decision. Details of the search can be found in Appendix.

### 2.3 Study selection

After removing duplicate literature from the search records, all retrieved literature was independently reviewed for abstracts and full texts by researchers (Lu and JH Huang) at the same time using the same review scheme. The reference lists of included articles and related review articles were manually searched. In the process of full-text review, if there is any disagreement, it will be reviewed by a third researcher (HY Huang), and the final result will be decided by the three together.

The inclusion criteria were as follows: (1) The scope of the included patients is stroke patients without neurological diseases or reported TMS contraindications; (2) The intervention group must use iTBS to intervene with the subjects; (3) The included literature must include at least one of the following outcome indicators: upper extremity Fugl-Meyer Assessment (UE-FMA), Action Research Arm Test (ARAT) or Barthel Index (BI); (4) The experimental design of the study must be a randomized controlled trial.

Exclusion criteria: (1) unable to extract valid experimental data from the study; (2) Not only focusing on the recovery of upper limb function, as it may be difficult to determine the independent effect of iTBS on the upper limb in joint studies;(3) Did not report the mean and SD values of the changes in the effect size calculation results;(4) They are review studies, case reports, conference reports, or abstracts;(5) Research published in languages other than English or Chinese.

### 2.4 Data extraction and management

Two researchers (Lu and JH Huang) independently extracted data related to trial characteristics from the included studies using a standardized form. The extracted data includes: author and year of publication, country of the trial, age of the subjects, number of people in the intervention group and control group, details of the intervention and control conditions, the outcome of each trial examination, and data related to the mean and SD values of the outcome changes (i.e., changes from baseline to after intervention). After all data extraction was completed, the results were cross-checked, and all discrepancies were reviewed and corrected by the researcher (HY Huang).

### 2.5 Quality assessment

The risk of bias in the included studies was independently evaluated by two investigators, Lu and JH Huang, utilizing Review Manager 5.4. This objective assessment involved a thorough examination of the full text of each study. The evaluation encompassed several domains: selection bias (encompassing random sequence generation and allocation concealment), performance bias (pertaining to the blinding of participants and personnel), detection bias (related to the blinding of outcome assessment), attrition bias (due to incomplete outcome data), and reporting bias (stemming from selective reporting). Each of these domains was scrutinized and subsequently classified into one of three categories: (1) low risk of bias, (2) high risk of bias, or (3) unclear risk of bias.

### 2.6 Data analysis

The data incorporated in this study underwent analysis and processing utilizing the Cochrane Collaboration's Review Manager 5.4, a software designed for meta-analysis. In the included studies, the follow-up results conducted at different times of the trial were evaluated multiple times. We opted for results proximal to an 4-week duration for our analysis. Given that all the values measured were continuous variables, the Standardized Mean Difference (SMD) was employed for difference calculation. The 95% Confidence Interval (CI) was assessed using the *z*-test. The Cochran's *Q*-statistics and *I*^2^ test were used to examine the heterogeneity among groups. In instances where no heterogeneity was detected among the groups (*P* > 0.05 or *I*^2^ < 50% as indicated by the *Q*-test), a fixed-effect model was implemented. Conversely, if the *Q*-test results were significant (*P* < 0.05 or *I*^2^ > 50%), a random-effect model was utilized in the meta-analysis (25). In situations where standard deviations were not reported, they were computed based on standard errors, CI, or *t*-values. The *I*^2^ parameter was used to determine and quantify the statistical heterogeneity between each citation. An *I*^2^ value exceeding 50% was deemed as an indicator of substantial heterogeneity. A *P*-value < 0.05 was considered statistically significant.

## 3 Results

### 3.1 Search results

**Figure 1:**
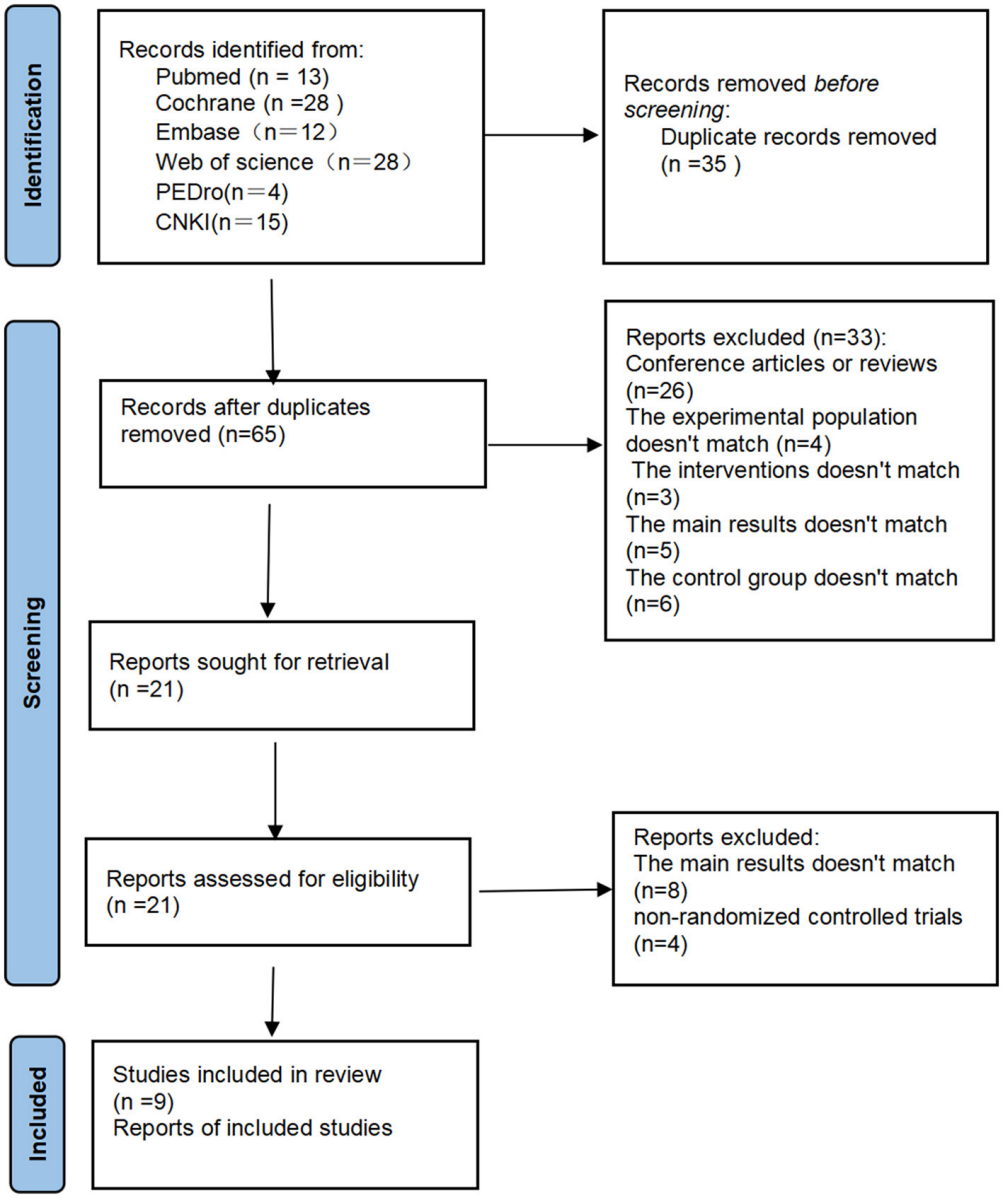
Flow chart of study selection.

After the initial literature search (PubMed = 13, The Cochrane Library = 28, Embase = 12, Web of science = 28, PEDro = 4, China National Knowledge Infrastructure = 15), a total of 100 articles were obtained. After removing duplicate literature, 65 articles were obtained. We made the literature screening process as Figure 1. All obtained literature was imported into EndNote for unified management. Among 65 the articles that underwent full-text screening for eligibility, 26 were deleted because they were conference abstracts or reviews, four were deleted because the trial population did not match, three were deleted because the control conditions were insufficient and did not meet the task requirements, five were deleted because the main results did not match, and six were deleted because the control group did not match. After excluding the above 33 articles that did not meet the requirements, the full text of the remaining 21 articles was reviewed. Among them, four were deleted because they were not randomized controlled trials, and another eight were deleted because the main results did not match. This study finally included nine studies that met the standards.

### 3.2 Study characteristics

Table 1 shows the age characteristics of the subjects in nine randomized controlled trials. All studies were published between 2013 and 2022. Seven articles were published in English, with one each from India (26) and New Zealand (27), and five from China (15, 16, 28–30). The other two articles published in Chinese (31, 32) were both from China.

**Table 1:** Characteristics of the included studies.

**References**	**State**	**Test types**	**Age (years) (M ±SD)**	**Experimental group (*N*)**	**Control group (*N*)**	**Intervention protocol**	**Control scheme**	**Intervention protocol intensity**	**Outcome**
Chen et al. (28)	China	RCT	• E: 52.9 ± 11.1 • C: 52.6 ± 8.3	11	11	80% AMT	Sham iTBS	10 times in 2 weeks (5 times/week)	FMA-UE, ARAT
Chen et al. (16)	China	RCT	• E: 54.36 ± 10.56 • C: 48.95 ± 9.63	12	11	80% AMT	Sham iTBS	15 times in 3 weeks. 15 times (1 time/day)	FMA-UE, ARAT
Hsu et al. (29)	China	RCT	• E: 56.8 ± 6.8 • C: 62.3 ± 8.5	6	6	80% AMT	Sham iTBS	10 times in 10 days (1 time/day)	FMA-UE
Ackerley et al. (27)	New Zealand	RCT	• E: 61 ± 14.75 • C: 71 ± 10.25	9	9	90% AMT	Sham iTBS	10 times in 10 days (1 time/day)	ARAT
Meng et al. (15)	China	RCT	• E: 55.3 ± 7.47 • C: 52.5 ± 13.51	10	10	Initially set at 60%RMT, increased by 5% daily, reaching a maximum of 80% RMT.	Sham iTBS	10 times in 10 days (1 time/day)	FMA-UE, BI
Khan et al. (26)	India	RCT	• E: 63.55 ± 12.67 • C: 64.60 ± 12.99	20	20	60% AMT	PT	12 times in 4 weeks (3 times/week)	FMA-UE, BI
Zhang et al. (30)	China	RCT	• E: 52.00 ± 6.39 • C: 52.93 ± 9.25	14	14	70% AMT	Sham iTBS	10 times in 3 weeks	FMA-UE
Zhou and Liu (32)	China	RCT	• E: 48.75 ± 7.63 • C: 55.64 ± 12.60	12	11	80% AMT	PT	10 times in 2 weeks (5 times/week)	FMA-UE, BI
Lian et al. (31)	China	RCT	• E: 60.00 ± 11.26 • C: 61.60 ± 13.17	15	15	70% RMT	PT	24 times in 4 weeks (6 times/week)	FMA-UE, ARAT, BI

E, Experimental group; C, Control group; FMA-UE, Fugl-Meyer Assessment-Upper Extremity; ARAT, Action Research Arm Test; BI, Barthel Index; AMT, active motor threshold; RMT, resting motor threshold.

In six studies (15, 26, 28, 29, 31, 32) physical therapy was performed while iTBS in the intervention group. One study (27) provided personalized upper limb functional exercises to the subjects while implementing iTBS. One study (15) conducted robot-assisted training for the subjects while implementing iTBS. One study (16) conducted Virtual Reality-based Cycling Training (VCT) as an adjunctive therapy for the subjects while implementing iTBS.

Four studies (16, 28, 29, 32) chose to set the iTBS stimulation intensity to 80% RMT, one study (26) chose a stimulation intensity of 60% RMT. One study (15) set the initial intensity of iTBS at 60% rMT and increased it by 5% rMT daily until 80% rMT unless the patient had discomforts like headache or nausea. One study (31) chose a stimulation intensity of 70% RMT. One study (27) chose a stimulation intensity of 90% RMT. Another study (30) chose a stimulation intensity of 70% AMT.

Eight studies evaluated upper limb function as an outcome indicator (15, 16, 26, 28–32), four studies (16, 27, 28, 31) evaluated upper limb motor function after stroke, four studies (15, 26, 31, 32) evaluated the ability to live independently. Although different assessment scales were used in some studies, data related to the same outcome measures were analyzed together.

### 3.3 Quality assessment

**Figure 2:**
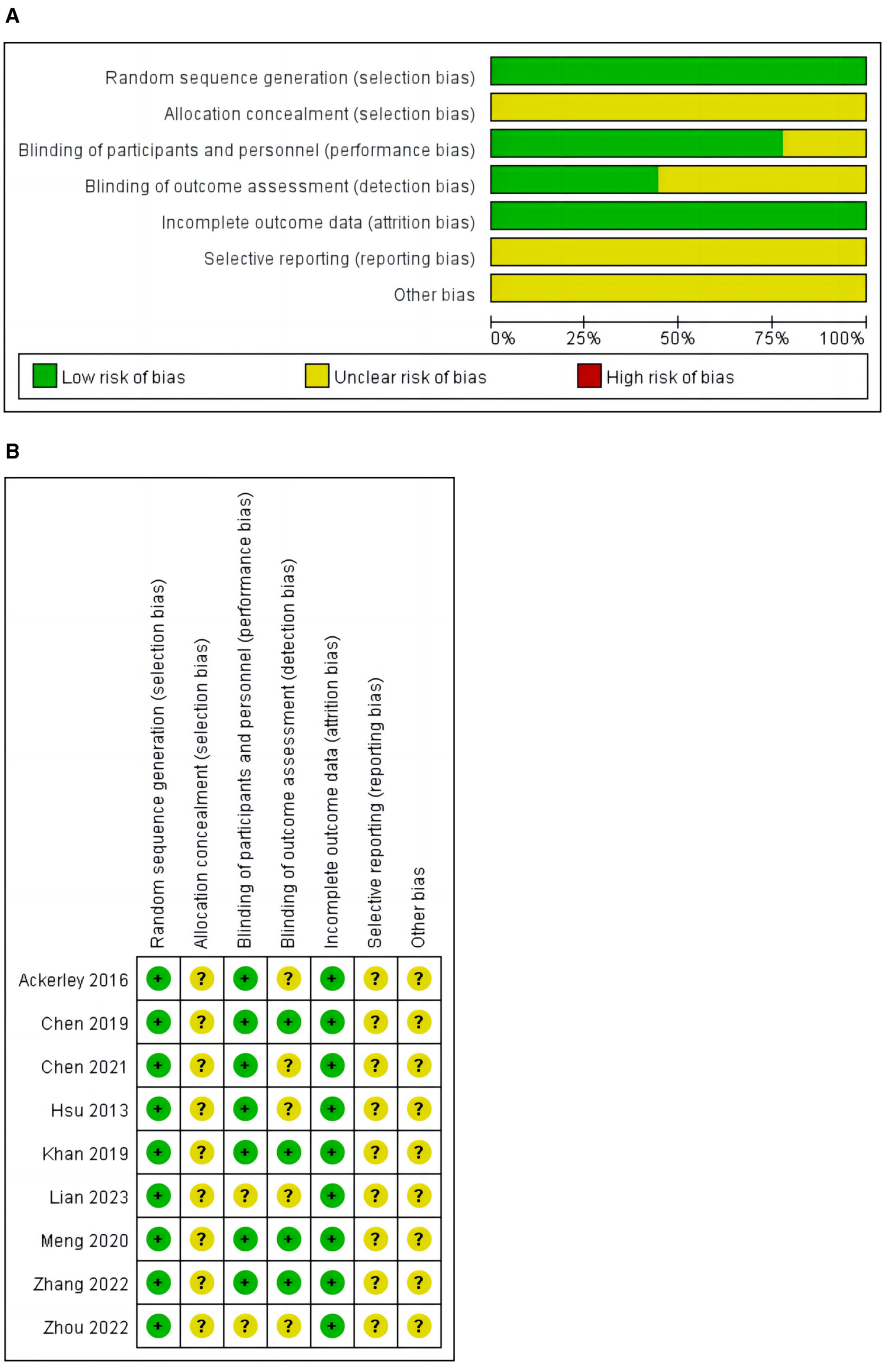
**(A)** Risk of bias graph. **(B)** Risk of bias summary.

A bias risk assessment was conducted on the nine included studies, as shown in Figure 2. All disagreements about bias risk domain ratings were resolved through discussions among all reviewers until 100% consensus was reached. The results are shown in Figure 2. All included studies were randomized controlled trials and clearly described their randomization methods in the articles (100%). No study explicitly stated that allocation concealment was implemented in the trial (0%). Seven studies (15, 16, 26–30) used blinding for participants and experimenters (77.8%). Four studies (15, 26, 28, 30) used blinding for outcome assessors and data analysts (44.44%). Incomplete outcome data (attrition bias) were all low risk (100%). After reviewing all included studies, the risk of selection bias was not clear (100%).

#### 3.3.1 iTBS on Fugl-Meyer assessment-upper extremity

**Figure 3:**
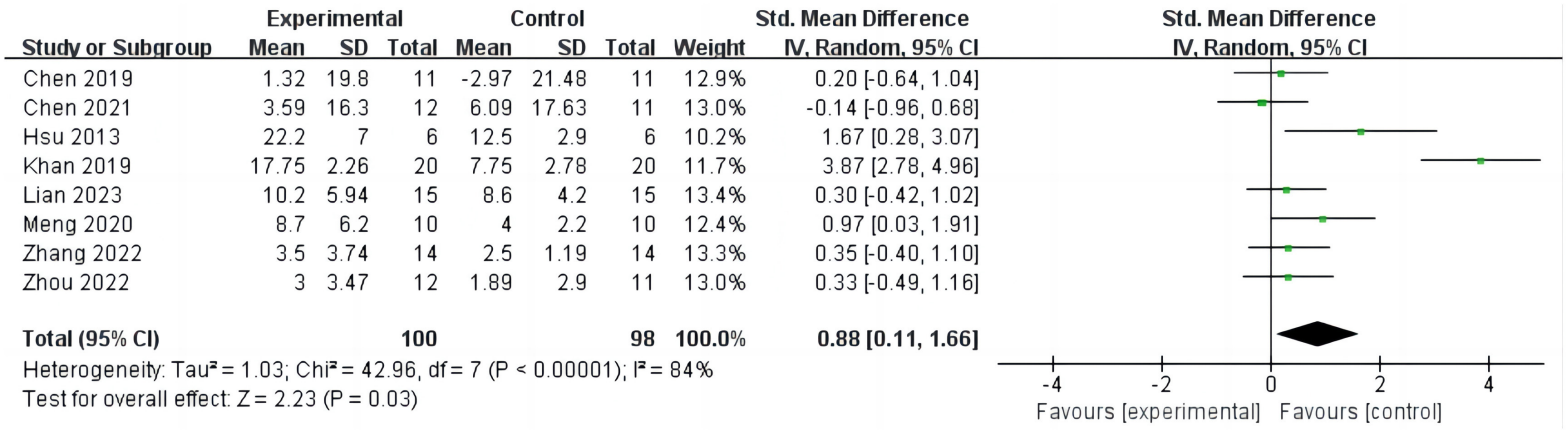
iTBS on FMA-UE

Eight studies (15, 16, 26, 28–32) reported the results of FMA-UE before and after intervention, involving 198 participants, as shown in Figure 3. The analysis results indicate that compared to the control group, iTBS effectively improves the FMA-UE scores of stroke patients (SMD = 0.88; 95% CI = 0.11–1.66; *P* = 0.03, *I*^2^ = 84%). Due to the high heterogeneity of the results, we conducted sensitivity analyses one by one and found that after excluding one study (26), the results still showed a significant improvement in upper limb function in stroke patients with iTBS intervention, although the heterogeneity was significantly reduced (SMD = 0.39; 95% CI = 0.05–0.73; *P* = 0.02, *I*^2^ = 9%).

#### 3.3.2 iTBS on Action Research Arm Test

**Figure 4:**
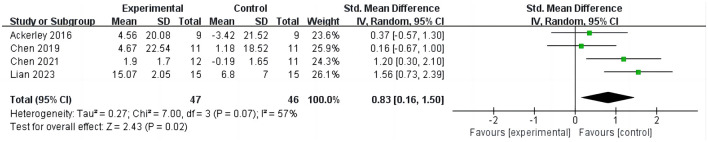
iTBS on ARAT.

Four studies (16, 27, 28, 31) evaluated the outcomes of ARAT in stroke patients, involving 135 cases, as shown in Figure 4. Four studies evaluated the outcome of ARAT in stroke patients, involving 135 cases, as depicted in Figure 4. The results indicate that compared to the control group, iTBS significantly improves ARAT scores in stroke patients (SMD =0.83; 95% CI = 0.16–1.50; *P* = 0.02, *I*^2^ = 57%). Due to the high heterogeneity, sensitivity analysis was conducted. Upon excluding one study (28), it was found that the ARAT scores in the iTBS group remained significantly higher than those in the control group, albeit with reduced heterogeneity (SMD =1.07; 95% CI = 0.38–1.75; *P* = 0.002, *I*^2^ = 44%).

#### 3.3.3 iTBS on Barthel Index

**Figure 5:**
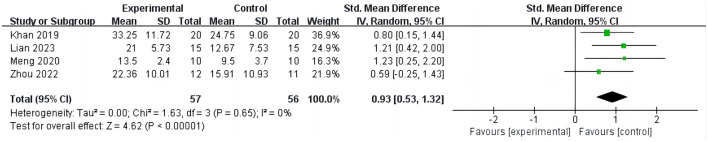
iTBS on BI.

Four studies (15, 26, 31, 32) involving 113 patients reported changes in BI scores before and after treatment in stroke patients as shown in Figure 5. The analysis results demonstrate that compared to the control group, iTBS significantly improves BI scores in stroke patients, with low heterogeneity observed in the results (SMD = 0.93; 95% CI = 0.53–1.32; *P* < 0.0001, *I*^2^ = 0%).

## 4 Discussion

Our systematic review and meta-analysis comprised nine RCTs, involving a total of 224 stroke patients, with the aim of investigating the impact of iTBS on upper limb function in stroke patients. To ensure the rigor of this analysis, we maintained the exact same inclusion criteria as those registered initially, fully aligning with the expected scope of work. Our research indicates that iTBS can effectively enhance upper limb function in stroke patients, improving their functional status in daily activities. This enables patients to independently perform essential life tasks, leading to a restoration of their quality of life to some extent. Our study results are largely consistent with previous research. However, unlike previous studies, we exclusively included RCTs to obtain more objective and accurate experimental data and results (33, 34).

### 4.1 Applications of other non-invasive brain stimulation techniques

rTMS is one of the earliest transcranial magnetic stimulation techniques. Its research has extensively covered various domains including depression, obsessive-compulsive disorder, and chronic pain. Studies (35) have shown that rTMS is particularly effective in treating depression, especially under conditions of repeated high-frequency stimulation. Additionally, rTMS demonstrates overall positive outcomes in the management of chronic pain (36), although individual variability and tolerance issues still require further investigation.

Transcranial Direct Current Stimulation (tDCS) is a low-cost and user-friendly neuromodulation technique employed in the treatment of various neurological disorders (37). Research (38) indicates that tDCS can enhance language functions in stroke patients. However, data on its long-term efficacy and underlying mechanisms remain limited. Additionally, there is currently no evidence demonstrating that tDCS improves upper limb function in stroke patients.

Previous meta-analyses have shown that iTBS demonstrates potential in improving upper limb function following stroke. Some studies report statistically significant effects of iTBS on enhancing upper limb motor function, particularly in acute and subacute patients (39). However, these studies often suffer from small sample sizes and high design heterogeneity, which impacts the generalizability and reliability of the conclusions (33). Although iTBS shows positive effects on improving upper limb function, current research predominantly focuses on short-term outcomes, and the long-term effects and underlying mechanisms remain unclear (33).

### 4.2 The effect of iTBS on upper limb function in stroke patients

Our analysis reveals that iTBS significantly improves upper limb function in stroke patients, as evidenced by a significant increase in FMA-UE and ARAT scores in the iTBS group compared to the control group. In our analysis of post-treatment FMA-UE scores, we found that although the iTBS group had significantly higher scores compared to the control group, there was high heterogeneity. Sensitivity analysis revealed that the source of this heterogeneity may stem from one study (26) that utilized a lower intensity of iTBS (60% of active motor threshold). After excluding this study, the heterogeneity markedly decreased. Furthermore, our findings revealed a significant enhancement in FMA-UE scores among stroke patients when compared with the control group. Therefore, we hypothesize that a higher intensity of iTBS (70–90% of the active motor threshold or resting motor threshold) may confer greater benefits for upper limb functional recovery in stroke patients (40, 41).

In the analysis of ARAT scores among stroke patients, results akin to those of the FMA-UE analysis were observed, wherein the ARAT scores in the iTBS group were significantly higher than those in the control group. However, we encountered a noteworthy issue of high heterogeneity. Sensitivity analysis indicated a reduction in heterogeneity upon excluding a study (28), and the final results remained statistically significant. Upon reviewing the entire manuscript, we discovered that, in addition to administering iTBS therapy, patients in the intervention group underwent a 60-min session of virtual reality-based cycling training on the same day following completion of the treatment. This novel combined therapeutic approach may be the underlying cause of the observed heterogeneity. Consequently, future experimental designs should account for the impact of this newly introduced combined therapy on trial outcomes.

Upper limb functional impairment has long been a significant issue for stroke patients (42, 43). FMA-UE is a commonly used clinical tool for evaluating upper limb function in stroke or other neurological injury patients (44). This assessment comprehensively evaluates multiple dimensions, including flexibility and coordination of the shoulder, elbow, wrist, and fingers. Following iTBS treatment, the patient demonstrated a significant improvement in FMA-UE scores, indicating enhancements across various functions of the upper limb joints. As a form of brain stimulation technique, iTBS can improve brain function by stimulating the cortical regions of the brain using magnetic fields (45). This improvement may be associated with the promotion of neuroplasticity in the cortical areas, including the formation and strengthening of synaptic connections, as well as enhanced communication between neurons (46). Such enhanced neuroplasticity facilitates the reorganization and reconstruction of damaged neural networks in the brain, thereby improving upper limb function (47). iTBS also benefits healthy brain regions. Following a stroke, brain areas surrounding the lesion may participate in compensatory motor control (48, 49). By stimulating healthy brain regions surrounding the lesion with iTBS, it can promote their activity, thus enhancing the effectiveness of compensatory motor control. iTBS not only controls upper limb activities in the cerebral cortex but also enhances the efficiency of neural signal transmission by modulating neuronal conduction and excitability (50, 51). This increases both the range and efficiency of upper limb movements.

The efficacy of iTBS therapy can vary depending on the intensity, frequency, and targeted areas of stimulation (19, 52). Different intensities and frequency adjustments of iTBS directly impact neuronal excitability and activation levels (53). While stronger stimulation may accelerate the rehabilitation process, it could also elevate the risk of neuronal fatigue and damage (54). Hence, when determining treatment parameters, individual patient circumstances and the severity of the condition must be considered to ensure safety during therapy. The selection of stimulation sites is crucial as it determines the specific scope of influence. Different site selections may affect related motor control areas, thereby yielding varied therapeutic outcomes.

### 4.3 The effect of iTBS on BI

The results of this study demonstrate a significant improvement in BI scores among patients receiving iTBS treatment compared to the control group post-stroke. The BI score reflects the patients' ability to perform activities of daily living, suggesting that iTBS therapy has a more effective role in the rehabilitation of daily living activities in post-stroke patients (55). The improvement of upper limb function in stroke patients is of paramount importance for their rehabilitation and enhancement of quality of life. Therefore, the improvement of upper limb function in stroke patients is of paramount significance for enhancing their independence and quality of life (56, 57).

The improvement in upper limb function enables patients to perform various tasks more effectively and independently in their daily lives. For instance, they can more effortlessly self-administer utensils and grooming items, dress independently, thereby reducing dependence on others (58). These actions contribute to enhancing the patient's autonomy and self-esteem. The enhancement of independence may positively impact the psychological wellbeing of patients, aiding in reducing issues like (59) and anxiety (59, 60). Improved upper limb function also offers patients greater opportunities for social interaction and engagement. They can communicate more freely with others, participate in community activities, and even reintegrate into work or volunteer service (61). This social engagement not only enhances patients' life satisfaction but also contributes to improving their sense of social integration and interpersonal relationships (62, 63). By facilitating the rehabilitation of daily living activities, stroke patients can better adapt to the demands and challenges of daily life. This enhancement in adaptability and coping skills equips patients with greater resilience to life stressors and challenges, enabling them to better adjust and integrate into society (64). Therefore, iTBS therapy can provide robust support and impetus for comprehensive rehabilitation and reintegration into normal life for stroke patients.

## 5 Study limitations

1. Due to limitations in the number of studies available, the final inclusion of research data did not allow for assessments of patients' psychological wellbeing or quality of life. This underscores the necessity for future research to involve larger sample sizes to ensure the objectivity of results and comprehensive assessment. 2. The intervention measures in the control group were not uniform, making it difficult to ascertain their impact on the outcomes, thus resulting in increased heterogeneity in this study. 3. In the included studies, the duration, frequency, and timing of interventions varied, as did the location of strokes. Patients' responses to iTBS therapy may be contingent upon the location and severity of stroke-related damage.

## 6 Conclusions

The results of this study indicate that iTBS serves as an effective therapeutic approach for improving upper limb function in stroke patients, facilitating enhanced capabilities for activities of daily living and autonomy. Future research should delve deeper into various combined iTBS therapies to elucidate their mechanisms of impact on upper limb function in stroke patients, thereby identifying optimal treatment strategies for enhancing upper limb function in stroke patients.

## Data Availability

The original contributions presented in the study are included in the article/Supplementary material, further inquiries can be directed to the corresponding authors.
